# 3D Bioprinting of Functional Skin Substitutes: From Current Achievements to Future Goals

**DOI:** 10.3390/ph14040362

**Published:** 2021-04-14

**Authors:** Paula Gabriela Manita, Itxaso Garcia-Orue, Edorta Santos-Vizcaino, Rosa Maria Hernandez, Manoli Igartua

**Affiliations:** 1 NanoBioCel Research Group, Laboratory of Pharmaceutics, School of Pharmacy, University of the Basque Country (UPV-EHU), Paseo de la Universidad 7, 01006 Vitoria-Gasteiz, Spain; paulagabriela.manita@ehu.eus (P.G.M.); itxaso.garcia@ehu.eus (I.G.-O.); edorta.santos@ehu.eus (E.S.-V.); 2 Bioaraba, NanoBioCel Research Group, 01006 Vitoria-Gasteiz, Spain; 3 Biomedical Research Networking Centre in Bioengineering, Biomaterials and Nanomedicine (CIBERBBN), Institute of Health Carlos III, 28029 Madrid, Spain

**Keywords:** skin bioprinting, 3D bioprinting, wounds, bioinks, tissue engineering

## Abstract

The aim of this review is to present 3D bioprinting of skin substitutes as an efficient approach of managing skin injuries. From a clinical point of view, classic treatments only provide physical protection from the environment, and existing engineered scaffolds, albeit acting as a physical support for cells, fail to overcome needs, such as neovascularisation. In the present work, the basic principles of bioprinting, together with the most popular approaches and choices of biomaterials for 3D-printed skin construct production, are explained, as well as the main advantages over other production methods. Moreover, the development of this technology is described in a chronological manner through examples of relevant experimental work in the last two decades: from the pioneers Lee et al. to the latest advances and different innovative strategies carried out lately to overcome the well-known challenges in tissue engineering of skin. In general, this technology has a huge potential to offer, although a multidisciplinary effort is required to optimise designs, biomaterials and production processes.

## 1. Introduction

In today’s society, most of the medical effort is focused on conditions, such as heart disease or cancer, which are considered top causes of death. However, other conditions, which may not be directly associated with death, yet bring about impaired quality of life and suffering, are also a crucial challenge for public health systems. A relevant example would be skin injuries and trauma, such as burns and chronic wounds, since the number of affected patients and the impact on the medical system is considerable. Chronic wounds, for instance, have a profound effect on quality of life [1]. In fact, pressure, diabetic and venous ulcers not only have an important impact on medical system expenditure—their economic spending is estimated to increase to more than 25 billion US dollars per year [1,2,3]—but also represent a burden for more than 7 million affected patients. In Europe, there are around 2 million patients suffering from chronic wounds, and in countries like the United Kingdom, the treatment of chronic wounds represents around 3%—5 billion pounds annually—of total health system costs [1].

Likewise, burns are also amongst the most common types of trauma worldwide. Indeed, burn-related medical attention is needed by 11 million people each year [4]. Approximately 10% of those burn injury patients present burns covering 30% or more of their total body surface. The mortality is substantially high among those patients, and, furthermore, the survivors are left with lifelong disabilities and disfigurement [5].

To understand why different scaffolds have been developed, we must first understand the basic pathophysiology of skin injuries. An ordinary cutaneous healing response consists of four distinct but overlapping phases: haemostasis, inflammation, proliferation and maturation [6]. During the haemostatic and inflammatory phases, blood coagulates, providing a shield, while blood flow to the wounded area is increased, allowing extravasation of plasma, generation of fibrin matrix and invasion of immunocompetent cells to clean the tissue. These cells, namely macrophages, with the aid of mesenchymal stem cells, attracted during the inflammatory phase, are responsible for the activation of fibroblasts and vascular endothelial cells. Subsequently, the proliferative phase begins, and the fibrin matrix is replaced with collagen, produced and secreted by fibroblasts. At the same time, angiogenesis is promoted in the granulation tissue, and keratinocytes migrate from the wound edges to the surface, while re-epithelisation begins [6,7,8]. Finally, during the maturation or remodelling stage, the newly formed dermis regains its strength [9,10]. This series of chronologically arranged but overlapping steps are carefully regulated by cytokines and different growth factors [11,12,13,14].

Although replacing the missing skin with healthy tissue from the donors, themselves (autograft), or a donor (allograft) remains the gold standard treatment for skin injuries, grafting is not a solution as simple as it seems. On the one hand, the patient may not have enough skin available for grafting—e.g., after extensive burns—and a donor site wound creation may not be recommendable. On the other hand, allografts coming from living donors or cadavers may be rejected by the patient’s immune system or may cause the transmission of viruses, such as hepatitis B or C or Human Immunodeficiency Virus [6,15,16]. Additionally, the existing shortage of donors worldwide must also be taken into consideration [17].

Taking all this into account, it is reasonable that new treatment strategies aim to replace the damaged skin tissue and to enhance the production of new skin constituents, such as skin cells, extracellular matrix, vasculature and skin appendages, to accelerate the wound-healing process [9,18,19]. In the last 30 years, advances in tissue engineering have allowed the development of alternatives to skin grafts, known as skin substitutes. There is a broad range of them already commercialised, which can be mono- or bilayered, cellular or acellular, biologic or synthetic [6,18,20,21,22]. The first developed scaffolds were acellular and consisted of extracellular matrix (ECM) components [23], collagen being the most commonly used material—e.g., bovine type I collagen—[23,24,25]. Products, such as Integra^®^, composed of a bilaminate sheet of cross-linked bovine tendon collagen and shark glycosaminoglycans with a silicone sheet cover, and Biobrane^®,^ which consists on a bilaminate membrane of semipermeable silicone membrane bonded to a layer of nylon mesh coated with porcine type 1 collagen, are examples of such acellular synthetic scaffolds [21,26,27,28]. Some wound care devices were synthesised using decellularised cadaveric dermis, a more complete ECM that could provide cells with a better mechanical support and promote their migration [18]. Examples of these decellularised allogenic dermis scaffolds are GraftJacket^®^, composed of cryopreserved cadaveric dermal collagen, and Alloderm^®^, an allogeneic lyophilised cadaveric collagen skin substitute [21]. Taking a step further, human placental membrane products have been developed, which are obtained from healthy female donors during routine caesarean section deliveries. The commercially available placental allografts can be bilayered, containing both the amnion and chorion layers of the placenta, or monolayered, containing only amnion products. Among bilayered products, there is Amnioband^®^, an aseptically processed, dehydrated human amnion and chorion allograft, and among monolayer products is Amnioexcel^®^, a dehydrated amniotic membrane allograft [29,30,31]. Those skin substitutes are clinically used for the treatment of chronic wounds, such as diabetic foot ulcers or leg ulcers, and for the treatment of superficial to extensive burns [21,26,27,28].

More recent products have taken these approaches one step further by housing skin-derived cells and growth factors within the scaffolds, with the aim of replicating the skin’s physiological structure. For example, some therapeutic approaches comprise a lower layer of fibroblasts representing the dermis and an upper layer of keratinocytes as an epidermis. This design confers similar mechanical properties to those of natural skin and, furthermore, would also recapitulate the paracrine function of skin cells to promote a faster and more efficient healing of the damaged tissue [32,33,34]. In particular, it would act as a sustained delivery system of growth factors, which are key factors in wound healing regulation, and their exogenous administration has been proven to accelerate healing [35]. Examples of cells containing skin substitutes would be Apligraf^®^, bovine type I collagen seeded with allogeneic neonatal foreskin fibroblasts and keratinocytes; Dermagraf^®^, synthetic polyglycolic or polylactic acid (PGA orPLA), ECM and allogeneic neonatal foreskin fibroblasts; or GRAFIX-Prime (viable cryopreserved placental membrane), which are clinically indicated for chronic wounds, such as diabetic foot ulcers, and partial and full thickness burns [36,37,38,39,40].

Even if there is some evidence that existing cell-containing skin substitutes provide advantages in wound healing over acellular formulations [41], there are also studies that question these cellular products’ cost-efficacy. In a comparative study carried out by Zelen et al., acellular skin substitute Oasis^®^ reported similar efficacy to Apligraf^®^, achieving complete wound closure within 4–6 weeks at a considerably lower cost [42]. Another study compared Theraskin^®^ and Apligraf^®^. Despite reporting no statistically significant differences in the healing rates, the expenditure using Apligraf^®^ was 42.2% lower [43].

There are still several challenges tissue engineering must overcome to achieve a fully functional artificial skin product [33,34,44], starting with the simplified bilayered structure. The more accurate the microarchitecture of the artificial skin, the better cell–cell, cell–matrix and dermo–epidermal interactions become, thus allowing a faster healing process and tissue recuperation, as well as having better mechanical properties. However, conventional methods of scaffold fabrication, such as electrospinning, fibre deposition, hydrogel casting, freeze drying or gas–foaming, lack strict control of structural features, making it impossible to recreate the skin’s complex structure and, consequently, its functionality [45]. The second shortcoming is the fact that skin substitutes fail to address the need of neovascularisation in the wounded area [46]. An insufficient blood supply compromises the amount of oxygen and nutrients available for cell division and interaction through cytokine production, slowing down the healing process and leading to rejection of the implanted skin substitute. In a nutshell, for cell-laden bioscaffolds to become a more clinically relevant substitute of skin grafts, a novel fabrication method is required that overcomes both poor perfusability and lack of anatomical accuracy [3,47,48].

In this context, the emerging technology of 3D bioprinting (3DBP) has been turning heads as a possible solution [46]. Three-dimensional BP is an advanced manufacturing platform that enables the predefined deposition of biomaterials, living cells and growth factors by means of computer-aided design (CAD). In this manner, it is possible to fabricate custom-designed tissue constructs by an additive manufacturing—layer-by-layer—printing process with a high degree of flexibility and repeatability [49]. This allows creation of complex, heterocellular structures with anatomical precision and provides control over different parameters essential for promoting cell adhesion and migration—e.g., pore size, interconnectivity and density of ECM—whilst maintaining good cell viability [46,50]. Moreover, bioprinting technology has been rapidly advancing over the past few years and its accessibility has never been greater [46,50,51].

## 2. Bioprinting: Principles and Techniques

There are different bioprinting techniques that have been used for the development of scaffolds. The common denominator is the computer-aided design that serves as a template to guide the bioprinting hardware into accurately patterned depositing of biomaterials [45]. The approaches described in the following paragraphs are successful tools for obtaining 3D-printed skin models.

### 2.1. Inkjet Bioprinting

**Figure 1:**
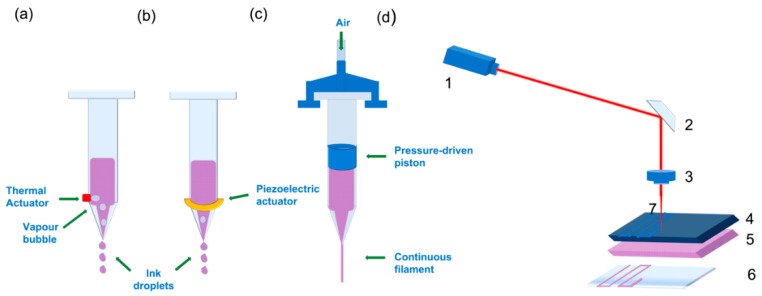
Schematic image of different bioprinting approaches: Two different types of inkjet droplet-based bioprinting approaches: (**a**) Thermal: The droplets are generated via a heating device that produces vapour in the thermal inkjet bioprinting approach. The bioink is ejected by deformation of the liquid cavity caused by the thermal action. (**b**) Piezoelectric inkjet bioprinting: the device responsible for generating the desired droplets is a piezoelectric actuator, also marked with a green arrow. In this case, the piezoelectric device increases its size, deforming the liquid cavity, and generates a droplet. In both cases, the cartridge is loaded with bio ink. (**c**) Pressure-assisted bioprinting. The green arrow is showing air entrance; the compressed air inside the cartridge forces the piston down onto the loaded bioink, and the movement of the piston extrudes the bioink through the nozzle in a continuous filament. (**d**) Laser-induced forwards transfer (LIFT) technique representation. (1) Laser source or diode. (2) Mirror. (3) Focusing lens. (4) Surface receiving laser irradiation. (5) Bioink containing cells. (6) Receiving substrate. (7) Laser beam. When (4) absorbs the laser beam, a vapour bubble is induced in (5), and a droplet encapsulating bioink and cells is created.

Inkjet printing is a noncontact printing technology based on desktop printers that reproduces the initial digital design onto a substrate with tiny ink drops; the resolution that can be achieved with this technique is about 20–100 µm [45,52]. Depending on what type of energy is used to create the drops and eject the bioink from the nozzle, inkjet bioprinting may be thermal (Figure 1a) or piezoelectric (Figure 1b) [45,52]. In piezoelectric inkjet bioprinting, ink is ejected through the nozzle with the use of an electric pulse, which causes the piezoelectric actuator to increase its size, pushing the ink droplet forwards. Although some biomolecules, like DNA, have been successfully printed, this approach is not optimal for printing living cells, since their viability may be significantly affected by the electric pulse [45,52]. On the other hand, thermoelectric inkjet-bioprinting has proven to be more biocompatible, because the biomaterial drop temperature only rises about 4–10 degrees above room temperature during printing.

### 2.2. Pressure-Assisted Bioprinting

Pressure-assisted bioprinting (PAB), also referred to as extrusion-based bioprinting, relies on a piston-driven, screw-driven or pneumatic force to push the ink through the nozzle onto the building platform in the form of a continuous filament with a maximum resolution of 200 µm (Figure 1c) [34,45]. Because of this printing mechanism, the cell-laden biomaterials used as bioinks for the production of 3D scaffolds must have certain rheological properties to ensure the flow of the bioink along the nozzle and its physical stability once printed. Although PAB allows less resolution in the obtained bioprinted scaffold (200 µm) compared to other bioprinting strategies, a wider range of biomaterials with different viscosities can be printed at room temperature [53,54,55,56,57].

### 2.3. Laser-Assisted Bioprinting

Both printing technologies described above involve the ejection of a bioink through a tiny nozzle. To avoid nozzle clogging or damage to cells due to shear stress at the orifice, the viscosity and cell density of the bioinks used is limited [58]. However, there are other approaches known as “orifice-free” that have overcome these drawbacks. One of the most popular approaches is laser-assisted bioprinting. This technique is based on laser-induced forwards transfer (LIFT) [59], which consists of a laser source irradiating a surface coated with the bioink of interest and a receiving substrate. When the laser irradiates the surface, a bioink droplet is created upon evaporation from the irradiated surface, and it is transmitted to the receiving surface (Figure 1d). This approach allows cell-level resolution—about 20 µm—and the possibility to print using higher cell densities, thus reducing the cultivation time of printed grafts, since printing techniques that don’t allow very viscous bioinks require postprocessing in order to obtain the desired cell density through cell proliferation. This only partly solves the problem, since production time is increased [3,45,59]. Moreover, published work support that the laser pulse does not harm living cells or promote stem cell differentiation in any way [58,60,61].

## 3. Bioink Choice

### 3.1. Biomaterials

**Figure 2:**
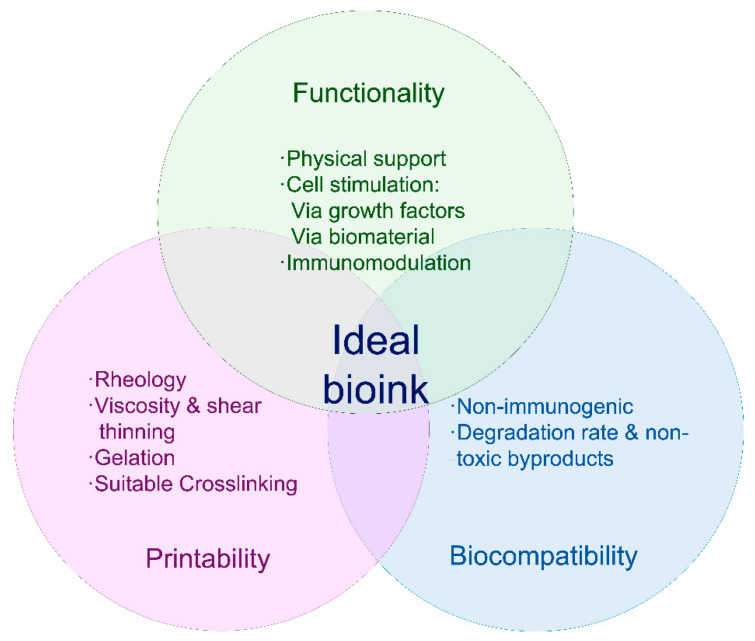
Requirements for bioinks. The ideal bioink must present the characteristics described in the figure above, and it should be suitable for the bioprinting device to be used, biocompatible and actively aid tissue reconstitution.

Bioink is the term used to refer to a biomaterial, usually a hydrogel with the desired cells embedded within it, ready for the bioprinting process [17]. In order to obtain a stable and accurately bioinspired printed construct, bioinks must be biocompatible and have certain rheological properties (Figure 2) [62].

Regarding the biomaterial form, hydrogels have been widely used in tissue engineering, thanks to their advantages. Their high-water content mimics natural tissue, and their porosity offers good permeability to oxygen and nutrients. These features create an environment that protects the cells during the bioprinting process and allows them to carry out their clinical function once the printed construct is applied to the damaged tissue [63,64,65]. In this context, hydrogel viscosity is also an important factor to consider. A more viscous hydrogel will remain in the desired shape once printed; however, increasing viscosity entails applying a higher shear stress during the printing process, which has been demonstrated to reduce cell viability greatly. A possible solution for this limitation would be immediate crosslinking of low viscosity hydrogels right after printing [66,67,68,69,70]. According to open literature, hydrogel viscosity should be aimed at 30–6 × 10^7^ mPa, whilst storage modulus in the final bioprinted construct is suggested to stand within 10^2^–10^3^ Pa [66].

Printability of a specific bioink mainly depends on rheological parameters, such as viscosity and shear-thinning, especially in inkjet-printed products. Gelation is also a key step, since the process must be fast so the printed filaments remain in the desired shape, yet not too aggressive, as it could affect the viability of cells embedded in the bioink [11,66,71]. Biocompatibility is also necessary. Ideally, bioinks must be nonimmunogenic and degradable at a rate similar to that of the targeted tissue recovery, and they should allow cell attachment and proliferation. Ideally, the biomaterial’s mechanical properties should also be skin-matching, and, regarding shape and structure, suitable porosity and homogeneity are required [62,72].

Due to the many requirements, choosing the right biomaterial for 3DBP of skin substitutes is a challenge that lies in finding the correct balance between biocompatibility and mechanical properties [62,73,74]. In general, biomaterials that provide good mechanical properties to the printed construct are not optimal for cell housing, whereas materials that simulate a tissue-like environment for living cell protection lack the physical characteristics needed to ensure printability and stability [62,73,74,75]. In any case, biomaterials can be divided in two main classifications: based on their source, they can be synthetic or natural. According to the characteristics of the materials they are composed of, the bioinks can be classified as structural, fugitive, support and functional [62]. These are summarised in Table 1.

Naturally occurring biomaterials are a popular choice in the field of tissue engineering, because of their resemblance to native ECM, linking them with optimal biological characteristics, such as biocompatibility, biodegradability and hydrophilicity. Most are proteins—e.g., albumin, collagen, thrombin, fibrinogen—or polysaccharides—e.g., chitosan, chitin, cellulose, alginate, hyaluronic acid. Nevertheless, besides poor mechanical characteristics, low reproducibility in their production process and elevated price are other drawbacks implied in the use of these biomaterials [100].

On the other hand, synthetic biopolymers like polyglycolic (PGA) or polylactic acid (PLA), polycaprolactone (PCL) or polylactic-co-glycolic acid (PLGA) are also popular in the field of bioprinting, due to their good mechanical properties [95,96,97,98]. However, the use of synthetic biopolymers also entails certain limitations, since they differ greatly from natural tissue and have poor or no cell recognition sites [100,101]. It is clear that no biomaterial by itself has the optimal requirements to be used as a bioink. Hence, the trend has been to combine biomaterials of different sources to overcome the setbacks encountered when used individually [44,66]. The choice of biomaterials should be such that it matches the mechanical properties of the target tissue and the requirements of the bioprinting device. However, it is printability that usually has the last say, when it comes to tuning bioink characteristics. In a general overview, we could highlight certain biomaterial combinations that have been used in multicomponent bioinks.

Alginate, a natural polymer extracted from seaweed, is a popular choice in bioprinting, since it is biocompatible and easily cross-linkable by ionic exchange with divalent cations at room temperature [66,67]. It also ensures protection to cells during the printing process [66,68]. Alternatively, given that it is a bioinert material, bioinks containing alginate are often functionalised by blending with other polymers, such as gelatin, which contains tripeptide Arg-Gly-Asp (RGD sequences), or fibrin to allow cell interaction with the bioink and enhance cell attachment and growth [66,68,69,70]. Another possible need of functionalising an alginate-based bioink would be with the aim of modifying its mechanical properties. Due to low viscosity, bioprinted alginate constructs tend to suffer from poor shape-fidelity [66,102]. In this regard, suspending cellulose nanocrystals in the bioink has proven to be a good strategy to confer shear-thinning properties and, thus, obtain a modified bioink optimal for printing [66,102,103,104].

Other combinations that have been proven successful are silk fibroin and gelatin [66,77,80], agarose and collagen [66,105] or chitosan and gelatin [89].

Mixtures of natural and synthetic polymers have also proven to yield functional bioinks. For example, alginate was added to polyethylene glycol diacrylate (PEGDA) in order to modify its printability. In this case, the mentioned mixture resulted in an increase of the bioink’s elastic modulus, from 5 kPa to 75 kPa [66].

The biomaterials used for bioink development described in this section act mainly as a structural component and facilitate the inclusion of cells and active biomolecules essential for tissue reconstruction, which will be discussed in the following section.

### 3.2. Bioink: Cellular and Biomolecular Component

Despite skin’s relatively simple layered primary structure, it is a complex tissue regarding different cell types; appendages, e.g., sweat grands or hair follicles; microvasculature; nerves and ECM components. Tailoring every single element into a bioink seems, at this stage of skin bioprinting, too difficult a task. Nevertheless, native skin tissue functionality and microarchitecture is achieved by endowing the 3D constructs with the capacity to self-assemble and develop, which is mainly done through the interactions of cells embedded in the bioink and their environment [33,89,106,107,108,109,110,111,112].

**Figure 3:**
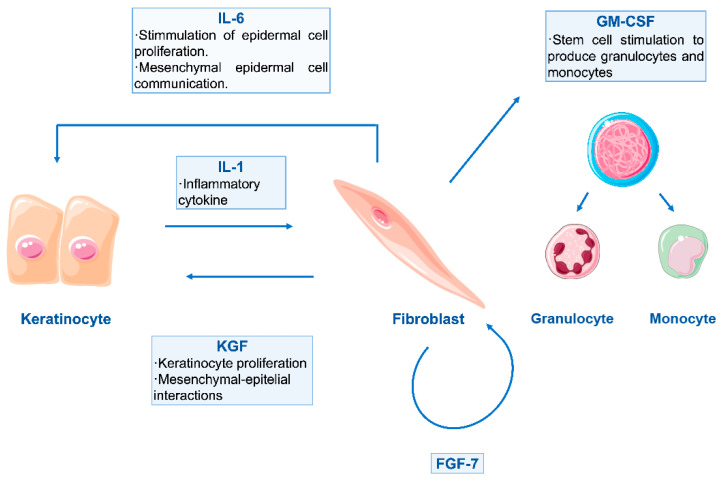
Schematic representation of double paracrine action between keratinocytes and fibroblasts. Keratinocyte-produced Interleukin-1 (IL-1) has an important role in this paracrine loop, as it targets fibroblasts and enhances the production of both fibroblast and keratinocyte growth factors (FGF–KGF). The production of Interleukin 6 (IL-6) is also initiated, and further stimulation of epidermal cells is given. Moreover, fibroblasts also release Granulocyte Macrophage Colony-Stimulating Factor (GM-CSF), in order to stimulate other cells for control and regulation of the wound healing process [116,118].

The choice of cells in bioinks has been mainly led by two native skin cell types: fibroblasts and keratinocytes [34,108,113,114,115]. Fibroblasts are distributed in the lower layers of the bioprinted skin construct, simulating a dermal compartment. Their function is to secrete glycosaminoglycans, proteoglycans and collagen, the main components of the ECM, giving structural integrity back to the damaged tissue. Superficial layers of the construct, destined to temporarily replace the missing epidermis, contain keratinocytes, which initiate the healing process and re-epithelisation. It has been demonstrated that the continuous crosstalk between these two cell types via cytokine and growth factor signalling is essential for correct wound healing, steering away from inflammation and into a state of cellular proliferation and synthesis [7,15,116,117]. An example of this interaction is shown in Figure 3.

However, these two types of cells are not the only ones present in healthy skin tissue. There are others which are also involved in wound healing and maintaining homeostasis. Mesenchymal stromal cells (MSC), naturally found within the pilosebaceous unit, are known for their immunomodulatory effect, fine tuning of the cytokine microenvironment and important role in correct scar formation [63,81,88,105,119]. Adipose Tissue-derived Mesenchymal Stem cells (AT-MSC) have also received attention, due to their regenerative potential and ECM remodelling functions [63,120,121,122,123]. Moreover, they have been found to promote neovascularisation through vascular epithelial growth factor (VEGF) secretion [63,121]. In this same context, different kinds of endothelial cells have been incorporated into 3D bioprinted scaffolds, yielding some degree of vascularisation [33,108,109,110,111,112]. Regarding pigmentation, melanocytes have also been incorporated successfully into bioinks [113,124,125]. Table 2 summarises the types of cells that have been used for printing skin constructs. The combination of the mentioned cell types in bioink formulations could help achieve a more physiologically relevant tissue (development of hair follicles, pigmentation, etc.) with better functionality. Generating more complex bioinks, in terms of cell types, also adds further difficulties to the existing formulation challenge, since finding media that will suit all cell types is not an easy task.

## 4. Advances in Skin Bioprinting

### 4.1. First Breakthroughs

The first bioprinted skin construct dates back to 2009, when pioneers Lee et al. employed a solid free-form fabrication system to produce a multilayered skin substitute containing superposed collagen precursor, rat-tail source type I collagen; human fibroblasts and human keratinocyte layers [83]. Their results, although only tested in vitro, showed good cell proliferation in both planar and nonplanar surfaces and certainly served as a boost to the idea of using 3D printing as an on-demand skin graft fabrication method. Later on, in 2011, Binder and collaborators were the first to evaluate their 3D inkjet-printed skin equivalents—human fibroblasts and keratinocytes in a fibrin and collagen matrix—in full-thickness wounds on athymic mice. They demonstrated the feasibility of wound healing by obtaining improved results over the controls—untreated, allogenic implant and hydrogel matrix [130].

In terms of technological development, inkjet and extrusion bioprinting techniques presented some drawbacks. Inkjet bioprinting only allowed printing inks with low cell densities to avoid high shear stress and clogging, and although this could be solved by using an extrusion method with a slightly larger diameter, it was at the expense of resolution [126,133]. When these drawbacks became well known, the scientific community then directed is efforts towards a manufacturing approach that would overcome the mentioned limitations in 3D printers. Against this background, the work of Koch et al. served as proof to demonstrate the advantages of laser-assisted bioprinting (LAB) [58,126]. The LAB printing procedure not only did not affect cell viability but also provided higher resolution in the printed construct and allowed printing at higher cell densities and any desired viscosity. Koch et al. used this printing technique to obtain a bilayered skin substitute composed of 20 sublayers of collagen–NIH 3T3 fibroblasts and 20 sublayers of collagen–HaCaT keratinocytes, stabilised onto a Matriderm^®^ sheet. To assess their product’s postprinted functionality, they relied on cadherin and gap–junction expression as an indicator of correct skin tissue development [58]. An in vivo wound healing study of this tissue engineered skin construct was carried out using dorsal skin-fold chamber approach. Although this method limits observation time, due to the chamber weight, it avoids wound contraction and prioritises re-epithelisation, which is the major healing method in humans. The results showed better vascularisation and tissue integration than the control construct maintained in vitro, since the in vivo implanted engineered constructs exhibited behaviours similar to those of physiological skin. However, as the experiment duration was short, keratinocyte differentiation was not clearly visible [134]. Despite laser-assisted bioprinting being a technological breakthrough in the tissue engineering field, its high cost is a main concern, since novel fabrication methods aim to provide reproducibility and high throughput at low cost [49].

**Figure 4:**
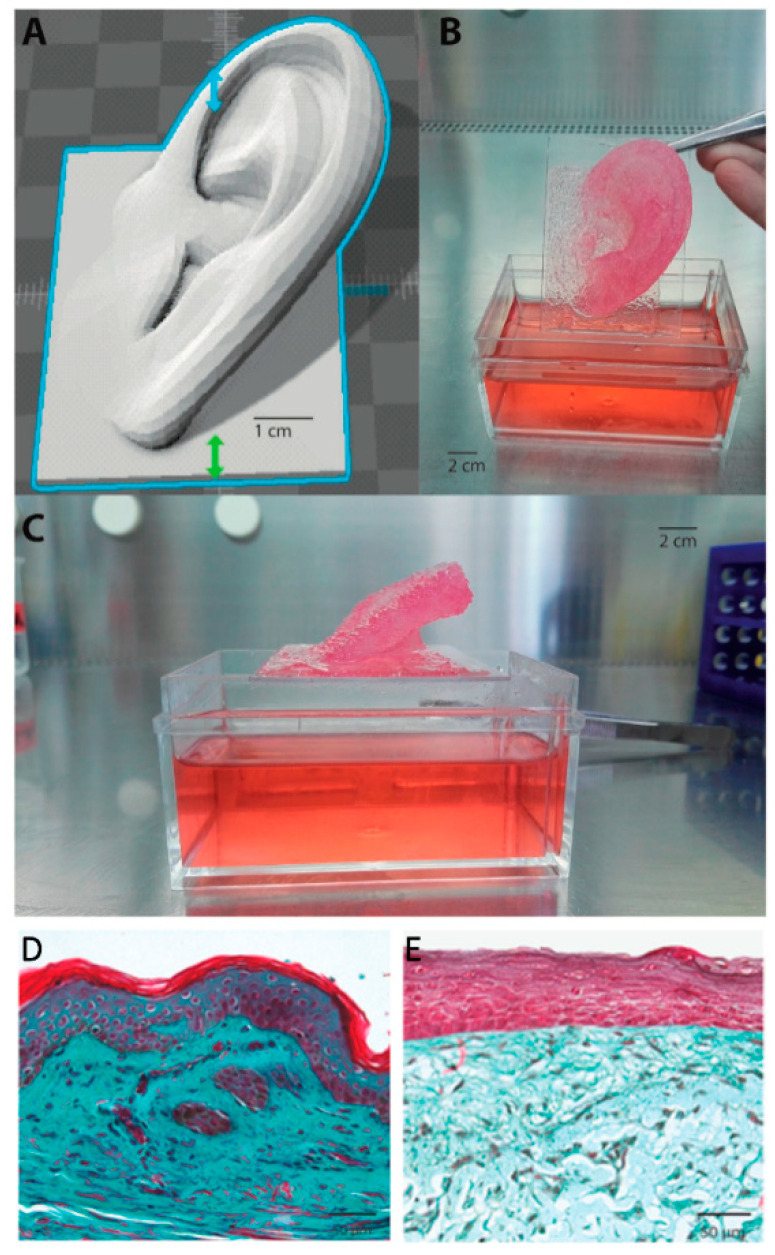
3D Bioprinted organ. (**A**) 3D computer-aided design (CAD). (**B**,**C**) 3D bioprinted adult-sized ear constituted of fibroblast containing dermal bioink. As can be seen, the complex architecture was retained during culture (i.e., immersion in Dulbecco’s Modified Eagle Medium (DMEM) culture medium). (**D**) Mason’s Trichrome staining of human skin. (**E**) Mason’s Trichrome staining of Pourchet’s bioprinted skin construct. Image reproduced with permission from Pourchet et al. [127]. Copyright 2017 John Wiley & Sons.

Advances have also been made in the characterisation and control of printing parameters in order to optimise printing processes. Lee et al. identified optimum parameters of air pressure, pulse duration, droplet volume and droplet spacing, as well as density, for both fibroblast and keratinocyte bioprinting. Moreover, they also describe a process of collagen gelation—they used a collagen matrix for bioprinting—by using aerosolised NaHCO_3_ as a cross-linker for collagen, which provided homogenous gelation and improved mechanical properties of the construct [33]. As mentioned before, progress in bioprinted tissue engineering products does not only depend merely on fabrication methods but also in the formulation of reliable bioinks. Novel strategies and compositions have also been worked on in recent years. In this context, Pourchet et al. developed a multifunctional bioink, which could maintain a suitable gel rheology during extrusion process, consolidate during postprocessing and allow a desired 3D network where tissue maturation could occur. This was obtained by using three different components. Gelatin was used as a rheological component and sacrificial material, which would be eliminated in subsequent steps; alginate to provide structure and the desired mechanical strength and fibrinogen for structure and cell-adhesion [127]. To finalise their study and present further proof supporting the feasibility of their bioink and technique, they produced a more complex bioprinted structure, using their dermal bioink and, subsequently, seeding keratinocytes. They also compared the histological appearance of their bioprinted skin against natural human skin. Although the bioprinted construct was able to support correct epidermal stratification and differentiation and was physiologically representative of human skin, a difference in Mason’s Trichrome colour could be observed in the dermal compartment (Figure 4). This was due to the fact that mature human skin possesses more ECM and a complex cocktail of cells, whereas the bioprinted skin only contained fibroblasts.

The previously mentioned work served as a proof of concept, demonstrating that 3DBP of cells and biomaterials is a good approach for producing synthetic biomimetic skin [87,115,126,130,134].

### 4.2. Vascularisation Strategies

**Figure 5:**
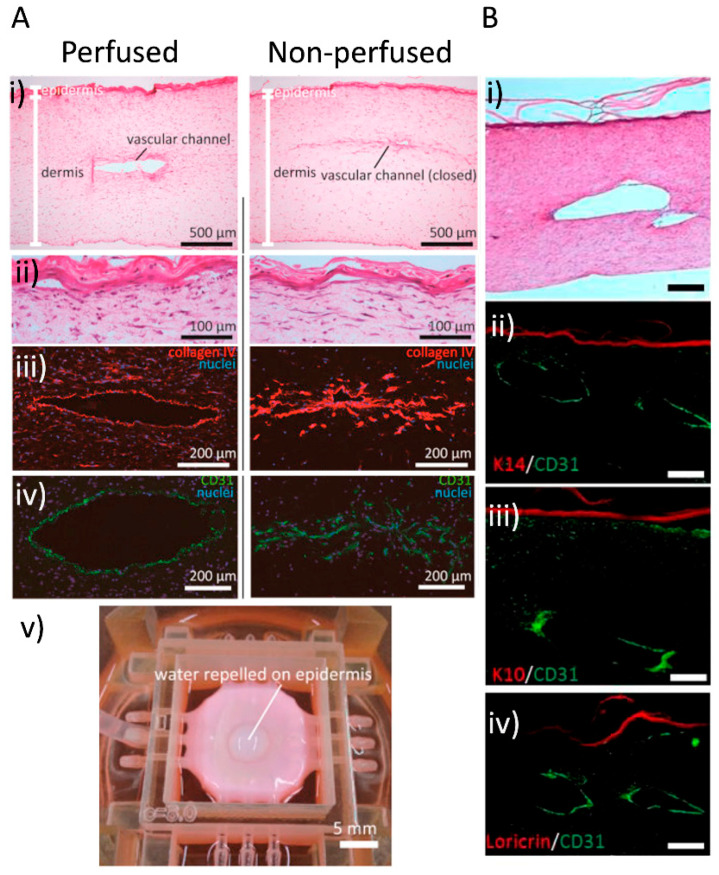
Vascular channels in bioprinted skin constructs. (**A**) Mori and colleagues’ work: skin integrated with perfusable vascular channels on a chip [109]. Histological analysis and barrier function assessment. Both perfused and nonperfused samples were cryosectioned for evaluation (i) Hematoxylin-eosin (H&E)-stained sections of perfused and nonperfused skin equivalents. (ii) Magnified images of the H&E-stained epidermal layers. (iii,iv) Immunostained vascular channels: (iii) Immunostaining with HUVEC marker CD31 and (iv) basal membrane protein (type IV collagen). (v) Barrier function evaluation by observing the repellence of water by the epidermal layer. (**B**) Abaci and colleagues’ work: human skin constructs with spatially controlled vasculature using primary and iPSC-derived endothelial cells. (i) Image showing H&E staining of the bioprinted skin equivalent showing normal skin physiology, and the lumen of the vascular channel is exposed. (ii,iii,iv) Immunofluorescent staining of histological sections of vascularised human skin equivalents generated using iPSC-derived endothelial cells. The sections were immunolabeled with K10, K14 and loricirin (red) and CD31 (green) to evaluate epidermal integrity and endothelial coating in the microchannels. Scale bars: 250 μm. Images reproduced with permission from Mori et al. and Abaci et al. [109,111]. Copyrights 2017 Elsevier; 2016 John Wiley & Sons.

Since this technology has been under the spotlight for over a decade, new challenges have emerged and, with them, different strategies to overcome them. One of the most important challenges is the need of a vascular network to ensure the transport of oxygen and nutrients, both to the printed cells in the graft and the healing tissue [53,135]. In 2014, the work of Lee et al. suggested that printed blood vessels could not replicate the function of natural blood vessels, primarily due to the many components and complicated microstructure of the latter [136,137]. Other 3D printing approaches involved micropatterning a perfusable vascular network within a skin construct [109]. Three-dimensional printing technology was used to print out moulds for sacrificial microchannels of cross-linked alginate that would remain embedded in the dermal compartment of the skin model (Figure 5). After keratinocyte differentiation and once the fibroblast and collagen layer had undergone contraction, the alginate was dissolved by perfusing a sodium citrate solution, and endothelial cells were seeded inside the channels. This resulted in a skin equivalent that was not only perfusable but also promoted neovascularisation, a property very much desired in the search for more physiologically relevant skin constructs.

The use of sacrificial materials has also been popular in attempts to overcome poor mechanical integrity in printed tissues and even organs. For example, Kang et al. developed an integrated tissue–organ printer. They incorporated microchannels to overcome the perfusion limit of 100–200 µm and sacrificial layers to retain shape, which allowed them to print tissues with higher complexity, as well as solid organs [114].

Another study describes the application of 3D printing to fabricate perfusable vascular channels coated with endothelial cells within a cultured skin equivalent. The skin construct was perfused via an external pump and tubes to provide nourishment. To assess their method, they ran a histological and cell-distribution analysis, which showed normal dermal and epidermal morphology, good barrier function and correct adhesion of the endothelial cells to the vascular channel wall, as seen in Figure 5. Absorption was also confirmed. These results put forward for consideration the idea of using perfusable skin constructs as models for systemic drug testing [111].

**Figure 6:**
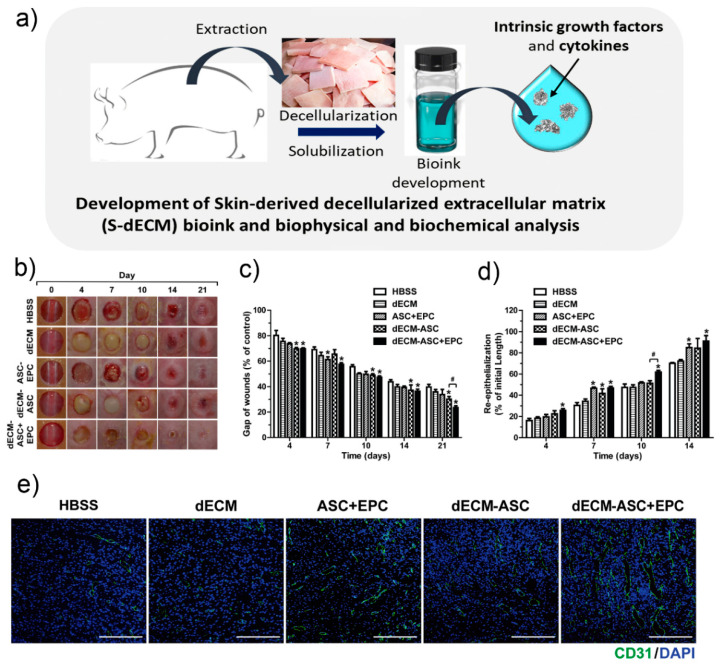
Kim et al.’s 3D cell printing of in vitro stabilised skin model and in vivo prevascularised skin patch using tissue-specific extracellular matrix bioink. (**a**) Development of decellularised extra cellular matrix bioink. (**b**) Representative photographs during 21 days of wound healing. (**c**) Variations of wounds gaps. (**d**) Re-epithelialisation values in the wounds areas on various days during wound healing. Data indicate mean ± SD. * *p* < 0.05 versus Hank Balanced Salt Solution (HBSS), # *p* < 0.05 versus dECM-ASC (skin equivalent composed of derived extracellular matrix and containing adipose-derived mesenchymal stem cells (*n* = 8–11). (**e**) In vivo vascularisation evaluation. Representative photographs of skin wound tissues on day 7 after immunostaining with anti-CD31 (green). Image reproduced with permission from Kim et al. [115]. Copyright 2018 Elsevier.

Another way to address the need for vascularisation and engraftment viability is through the development of bioinks that can provide more physical stability and tissue-specific microenvironment to cells, in addition to growth factors that promote vascularisation. For example, the work of Kim et al. presented a skin-derived extracellular matrix (S-dECM) bioink containing most of the ECM components, as well as cytokines and growth factors. When printing using this bioink, endothelial progenitor cells and AT-MSCs accelerated wound closure, and neovascularisation was observed. These data are shown in Figure 6. Despite proving their formulated bioink was more physiologically relevant than type I collagen bioink, they also noted that decellularisation methods needed to be improved, due to possible immunogenicity [115].

In a more recent work, Baltazar and collaborators were able to obtain a construct that was vascularised and biologically similar to human skin by incorporation of human foreskin dermal fibroblasts, human endothelial cells derived from cord blood endothelial colony-forming cells and human placental pericytes suspended in rat tail type I collagen into their dermal bioink. In their epidermal compartment, a second bioink formulation containing human foreskin keratinocytes was used. Rather than patterning and printing the actual vascular network, they observed that the endothelial cells and pericytes associated self-assembled into micro vessels in vitro. Moreover, in the presence of these cell types, keratinocyte maturation seemed to improve, since the implanted grafts were able to develop rete-ridges, a structure present in physiological skin histology that had been missing in previous studies [108].

### 4.3. Pigmentation

Since bioprinting of relatively simplified, cellular skin constructs has already been proved feasible, the objective is now to get closer to mimicking natural skin. One of the most basic features of natural skin is its pigmentation. Because of this, and with melanocytes being the main source of skin pigmentation, Min et al. decided to incorporate these cells into their biomimetic skin model. First, a dermal equivalent structure of collagen and fibroblasts was printed out, followed by the melanocytes, which were printed over the dermis in two different configurations, a 6 mm side square and 2 mm diameter single spots, the latter representing freckles [113]. Up to the date of their work, melanocytes added to scaffolds had not been able to produce visible signs of pigmentation or required activation through UV external stimuli [113,124,138]. In addition, Ng and co-workers put efforts towards facilitating the use of three different skin cell types when bioprinting. They identified the correct balance for a culture medium that would allow the proliferation and growth of fibroblasts, keratinocytes and melanocytes in co-culture. Moreover, the use of 3D printing technology allowed the design of hierarchical porous structures, mimicking those of natural skin, which contributed to the homogeneous distribution of the melanin granules produced in the epidermal region, yielding a naturally pigmented full-thickness skin graft [125].

In another recent experimental work, Jorgensen and collaborators produced a trilayered structure incorporating fibroblasts, keratinocytes, melanocytes, dermal microvascular endothelial cells, follicle dermal papillar cells and adipocytes, all suspended in a fibrinogen bioink [128]. The resulting tissue was implanted in athymic mice and harvested after 21 days for testing. Immunostaining revealed correct barrier function, dermal development, vascularisation and host cell integration. Moreover, wound healing occurred through re-epithelisation, unlike the case of the hydrogel group, where wound contraction was the primary mechanism seen. As a novelty, the group reported the formation of collagen basket bundles, compared to parallel fibres in hydrogel and untreated wounds after picrosirius red staining.

### 4.4. In Situ Bioprinting of Skin

**Figure 7:**
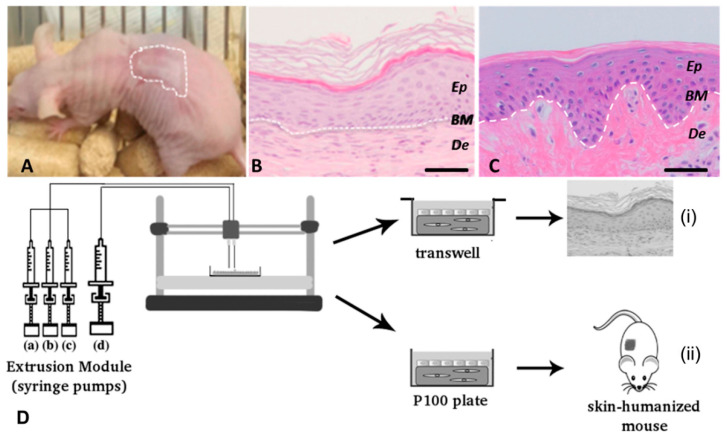
Cubo et al.’s 3D bioprinting of functional human skin: production and in vivo analysis. Histological analysis (8 weeks postgrafting) of bioprinted human skin grafted to immunodeficient mice. (**A**) Visual appearance of the grafted human skin. The dotted line marks the boundary between human and mouse skin. (**B**) H&E staining of the regenerated human skin. (**C**) H/E staining of normal human skin. The white dotted line in (**B**,**C**) indicates the dermo–epidermal junction (basal membrane, BM). Ep and De in (**B**,**C**) denote the epidermal and the dermal compartments, respectively. Scale bar: 100 μm. (**D**) Scheme of the bioprinting process. The extrusion module contained four syringes, loaded with human fibroblasts (hFBs) (a), plasma (b), CaCl_2_ (c) and human keratinocytes (hKCs) (d), respectively. The contents of the syringes (a–c) were continuously pumped out at the appropriate speed, mixed as they arrived at the head, extruded through the needle and deposited on the corresponding plate type (P100 or transwell), following the trajectories dictated by the control unit. This mixture was allowed to polymerise for 30 min at 37 °C to form a fibroblast-containing fibrin hydrogel, which became the dermal compartment of the skin equivalent. Immediately after this polymerisation step, the hKCs suspension contained in syringe (d) was similarly deposited on top of this hydrogel to form a confluent monolayer. (i) Equivalents printed on transwell inserts were allowed to differentiate at the air–liquid surface for 17 d and then analysed. (ii) Equivalents printed on P100 plates were grafted on to the backs of immunodeficient mice for eight weeks and then analysed. Reproduced with permission from Cubo et al. [129]. Copyright 2016 IOPscience.

Other important drawbacks associated with 3DBP technology include the need of specialised personnel, high production costs and the amount of time it takes to print out a clinically relevant skin construct [49,129]. Nevertheless, improvements in such aspects have been kicked off by Cubo et al. and, more recently, also by Quílez et al. These research groups have been able to print a skin substitute by combining human plasma, human fibroblasts (hFBs) and keratinocytes (hKCs) that was later on polymerised and grafted in less than 35 min, suggesting important potential for clinical applications [129,139]. In vivo analysis and the bioprinting process scheme is shown in Figure 7.

**Figure 8:**
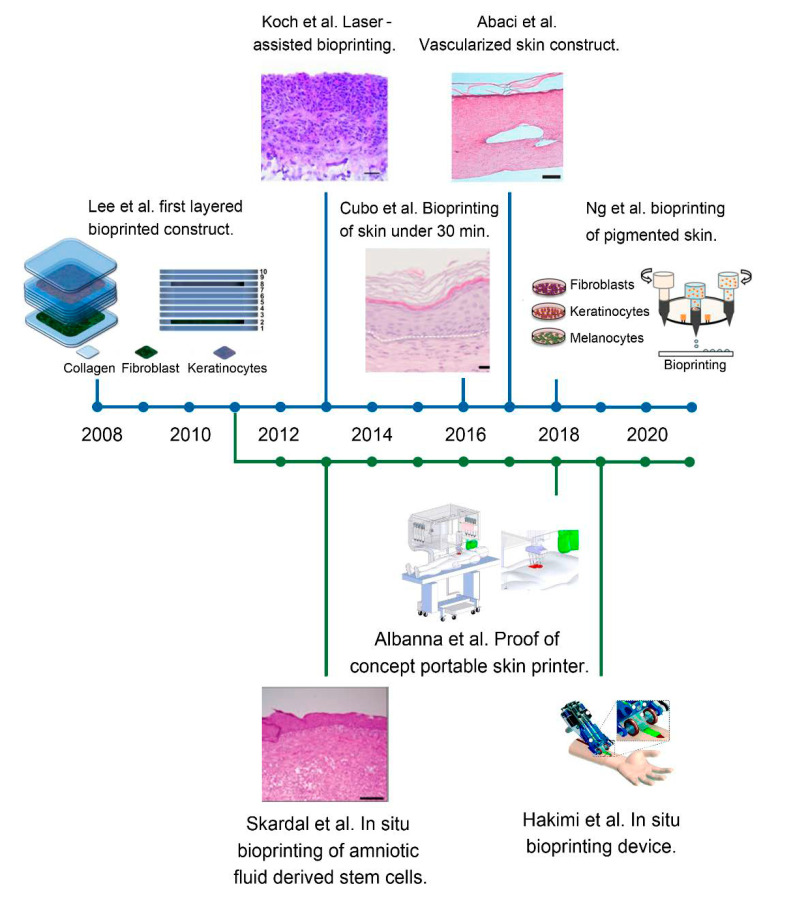
Timeline depicting the different advances and trends in bioprinted skin. The top blue line represents the in vitro approach, whilst the bottom parallel green line represents the in situ strategy. The evolution of bioprinted skin constructs is reflected, from the first simplified bilayered structures containing only two cell types, to the most recent work and notable achievements, such as the vascularisation, the inclusion of different cell types and the development of portable bioprinters. Reproduced with permission from Lee et al., Koch et al., Cubo et al., Abaci et al., Ng et al., Skardal et al., Albanna et al. and Hakimi et al. [81,82,83,111,125,129,131,132]. Copyrights2012, 2016 John Wiley & Sons; 2016, 2018 IOPscience; 2009 Elsevier; 2018 Royal Soci-ety of Chemistry; 2019 Nature-Springer.

Following the urge of quick and instant bioengineered skin constructs for immediate implantation, the idea of in situ bioprinting was born and started to develop independently of in vitro bioprinting, as depicted in Figure 8. This figure also summarises the whole development of 3D bioprinting of skin, from its first steps in 2008 and its upswing to come in the following years.

All the previously described studies involve bioprinting of skin in in vitro conditions, postprocessing and subsequent engrafting in animal models. However, real clinical application in patients could bring up potential complications, such as damage of the construct while transporting or manipulating and incorrect placement on the wound, which will most likely have a complicated 3D topology [3,49]. These issues could be avoided if the printing device allowed in situ bioprinting of the skin graft directly onto the patients’ wounds, regardless of the size, using their body as a bioreactor where the engrafted skin construct could functionally develop.

The first in situ bioprinting work was done in 2011 by Binder and collaborators, being also one of the first breakthroughs in bioprinting technology (see Section 4.1) [130]. Another important group developing advances in this area has been Skardal et al., who first printed amniotic fluid-derived stem-cells embedded in a fibrin–collagen bioink onto mice wound models [81]. Later on, they developed a tuneable hydrogel containing hyaluronic acid, which was designed to promote cytokine release [99]. Even if the stem cells in the first piece of work did not undergo permanent integration, their paracrine function was very much beneficial, as the release of trophic factors and cell–cell communication led to vascularisation enhancement and shorter wound closure time. On the other hand, Hakimi et al. developed a portable printer that allowed in situ formation of biomaterial and its deposition [131]. Albanna et al. also described a proof of concept of a portable skin bioprinting device that enabled the in situ imaging and precise deposition of cells and biomaterials onto extensive wounds. They applied this technology onto a porcine model, and it resulted in rapid wound closure and re-epithelisation in 8 weeks [132].

However, there are also disadvantages to this approach, as some bioinks used in tissue bioprinting require processes, such as chemical or UV treatment, to crosslink their materials and provide structural integrity, adding more complexity to in situ treatment of the patient [3].

## 5. Concluding Remarks and Future Perspectives

There is no doubt that 3DBP technology has a great potential to become a source of extremely precise, reproducible and fully functional synthetic organs. Nevertheless, despite the advances that have been described in this review, there are still many pending challenges on the road to develop a fully functional, clinically applicable and affordable 3D-printed skin constructs.

It is true that, as with any innovative technology, there has been an initial stage where expectations were unrealistically high, but 3DBP has already overcome that stage, and now problems are being successfully identified and tackled. To begin with, the complexity of any human tissue needs to be approached. To obtain a functional human organ, the bioprinted construct needs to contain multiple cell types embedded in different bioinks that confer adequate properties, hierarchical structure and interactions to each tissue. Perhaps that can be slightly more achievable in the case of 3D-printed skin, as its soft tissue and naturally layered structure make the design of the construct and deposition of biomaterials easier than with other organs. Bioprinters are not able to reproduce the structure of the skin yet, and, thus, they need to be upgraded, in terms of resolution, speed and number of channels available, to fulfil the requirements of skin tissue engineering.

Another crucial step related to bioprinters that needs to be overcome is the scaling up of 3D-printed skin manufacture to allow the treatment of large tissue defects and of a great number of patients. Some challenges that scaling up presents are achieving an acceptable resolution while printing large volumes of skin and the storage of produced skin substitutes, as they are living constructs. A possible alternative is to develop in situ bioprinters, which eliminates the necessity of scaling up. However, there are multiple technological issues to develop a bioprinter able to produce a functional skin construct in situ, along with logistical issues of storage of the bioinks containing living cells or issues related to the crosslinking process.

The convergence of different fabrication techniques can be helpful to overcome the technological problems that bioprinting of skin constructs presents. Using different bioprinting techniques or even adding other types of fabrication, such as electrospinning or casting, can bestow unique properties to the constructs, combining their advantages. For instance, the combination of laser-assisted and pressure-assisted bioprinting can achieve optimal resolution where needed, while being able to produce large volumes of construct in a faster way. Another benefit of convergence of techniques is the capacity to obtain layers with very different properties in the same constructs, due to the different fabrication methods. Finally, combining 3DBP with other techniques allows the use of biomaterials that cannot be included in a bioprinted skin construct, widening the range of possibilities to develop a skin substitute.

The development of an ideal bioink is also a challenge that needs to be addressed, for example, the obtention of a bioink with desirable porosity, viscosity, mechanical properties and ability to maintain cell viability. Regarding cell viability in skin constructs, although notable advances have been made in achieving some degree of vascularisation in the printed constructs, a challenge remains, to some extent, and the heterogeneity of experimental work suggests that the ideal approach to obtain vascularised tissue engineered skin has not yet been attained. The addition of different cell types to skin constructs is definitely a step forward to obtain more realistic products. However, obtaining skin annexes in 3D-bioprinted constructs, such as sebaceous glands and hair follicles, is a milestone which still seems far away. Even if melanocytes have been added to skin constructs to obtain pigmentation, the presence of melanocytes does not ensure a uniform pigmentation, and even less the obtaining of a pigmented skin construct that matches the skin tone of the patient, two challenges for the future that remain to be faced.

Innervation is an issue that has not been addressed yet in 3D-bioprinted skin constructs but represents an important objective to obtain a functional skin construct. Although there are multiple options to approach nerve regeneration, some strategies that have been followed to develop 3D-printed scaffolds for the treatment of neural damage include Schwann cells [140,141] or neural stem cells in the bioink [95,142], combined with dopamine-laden bioinks to enhance neural differentiation [143].

Accordingly, the ideal skin construct contains several cell types. However, there is a lack of information about the effect of these cells long-term. Safety studies are needed to dismiss the possible negative outcomes of the application of allogeneic cells, such as immune reactions, due to the breakdown of products; ectopic tissue growth, due to cell migration; and even the formation of teratomas, depending on the cell types [144].

Finally, to incorporate 3DBP into clinical practice, standardisation of the production process and regularisation are essential. The regularisation should include the tools, assays, markers, animal models and quality controls needed to ensure the safety and effectiveness of the skin constructs prior to their acceptance in clinical practice.

In all, if the scientific community makes a multidisciplinary effort to accomplish the aforementioned goals, 3DBP is undoubtedly on the way to becoming the next medical revolution.

## Figures and Tables

**Table 1:** Table summarising different bioink components according to their function and characteristics they provide in bioprinted tissues. (PCL: Polycaprolactone, PLGA: Poly lactic co-glycolic acid, GAGs: Glycosaminoglycans).

Function	Characteristics	Cells	Examples	References
Structural	They allow adhesion, proliferation and differentiation of printed cells, as well as cells from patient’s tissue [76,77,78,79,80].	Yes	Collagen	[81,82,83,84,85]
			Alginate	[68,69,84,85,86,87,88]
			Chitosan	[19,89,90,91,92,93,94]
Fugitive	Sacrificial materials that can be rapidly dissolved once their function is completed. Used strategically to create voids and channels within 3D structures.	No	Alginate	[84]
			Gelatin	[58,87]
Support	Usually synthetic materials used to provide physical strength and integrity.	No	Polyurethanes	[95]
			PCL	[85,96,97]
			PLGA	[98]
Functional	They influence cell behaviour and development through signalling and binding with growth factors.	Both	Heparins	[99]
			GAGS	[25,99]

**Table 2:** Table summarising the cell types used in the development of bioprinted skin constructs. (FB: fibroblasts, KC: keratinocytes, PAB: pressure-assisted bioprinting, LAB: laser-assisted bioprinting, HUVEC: human umbilical vein endothelial cells, iPSC: induced pluripotent stem cell, HECFCs: human endothelial cells cord blood human endothelial colony-forming cells, MC: melanocytes).

Cell type	Function	Source	Technique	Ref.
Fibroblasts	Cellular component of the dermis: secrete ECM components, giving structural integrity	Human dermal FB—neonatal and adult—NIH 3T3 FB Human foreskin FB Porcine dermal FB—autologous and allogeneic in a porcine animal model	PAB LAB In situ bioprinter	[33,58,83,84,108,109,111,113,115,125,126,127,128,129,130,131,132]
Keratinocytes	Epidermis component: initiate the healing process and re-epithelisation	Human epidermal KC—neonatal and adult—HaCaT cells Human foreskin KC Porcine epidermal KC—autologous and allogeneic in a porcine animal model	PAB LAB In situ bioprinter	[33,58,83,84,108,109,111,113,115,125,126,127,128,129,130,131,132]
Endothelial cells	Vascularisation	HUVEC iPSC-derived endothelial cells Endothelial progenitor cells HECFCs Human dermal microvascular endothelial cells	PAB Injekt bioprinting	[108,109,111,115,128]
Mesenchimal stem cells	Stimulate vascularisation and wound healing through growth factors and cytokines secretion	Adipose-derived mesenchimal stem cells Amniotic fluid-derived mesenchimal stem cells	PAB Injekt bioprinting In situ bioprinter	[81,99,115]
Perycites	Stabilisation of microvessels and regulation of vessel guidance in angiogenesis	Human placental pericytes	PAB	[108]
Melanocytes	Skin pigmentation	Human epidermal MC (neonatal and adult)	PAB	[113,125,128]
Preadipocytes	Help to modulate immune response and improve vascularization	Human hipodermis preadipocytes	PAB	[128]
